# Hyperbaric oxygen for patients with chronic bowel dysfunction after pelvic radiotherapy (HOT2): a randomised, double-blind, sham-controlled phase 3 trial

**DOI:** 10.1016/S1470-2045(15)00461-1

**Published:** 2016-02

**Authors:** Mark Glover, Gary R Smerdon, H Jervoise Andreyev, Barbara E Benton, Pieter Bothma, Oliver Firth, Lone Gothard, John Harrison, Mihaela Ignatescu, Gerard Laden, Sue Martin, Lauren Maynard, Des McCann, Christine E L Penny, Spencer Phillips, Grace Sharp, John Yarnold

**Affiliations:** a Hyperbaric Medicine Unit, St Richard's Hospital, Chichester, UK; b DDRC Healthcare, Plymouth, UK; c The GI Unit, The Royal Marsden NHS Foundation Trust, London, UK; d Clinical Trials and Statistics Unit at the Institute of Cancer Research, London, UK; e Whipps Cross University Hospital, Leytonstone, London & East of England Hyperbaric Unit, Great Yarmouth, UK; f London Diving Chamber, Hospital of St John and St Elizabeth, London, UK; g North West Emergency Recompression Unit, Murrayfield Hospital, Wirral, UK; h North of England Medical and Hyperbaric Services, Spire Hull & East Riding Hospital, Kingston-upon-Hull, UK; i The Diver Clinic, Poole, UK; j Division of Radiotherapy and Imaging, Institute of Cancer Research, London, UK

## Abstract

**Background:**

Hyperbaric oxygen has been used as a therapy for patients experiencing chronic intestinal syndromes after pelvic radiotherapy for decades, yet the evidence to support the use of this therapy is based almost exclusively on non-randomised studies. We aimed to provide conclusive results for the clinical benefits of hyperbaric oxygen in patients with chronic bowel dysfunction after radiotherapy for pelvic malignancies.

**Methods:**

HOT2 was a double-blind, sham-controlled, phase 3 randomised study of patients (≥18 years) with chronic gastrointestinal symptoms for 12 months or more after radiotherapy and which persisted despite at least 3 months of optimal medical therapy and no evidence of cancer recurrence. Participants were stratified by participating hyperbaric centre and randomly assigned (2:1) by a computer-generated list (block size nine or 12) to receive treatment with hyperbaric oxygen therapy or sham. Participants in the active treatment group breathed 100% oxygen at 2·4 atmospheres of absolute pressure (ATA) and the control group breathed 21% oxygen at 1·3 ATA; both treatment groups received 90-min air pressure exposures once daily for 5 days per week for a total of 8 weeks (total of 40 exposures). Staff at the participating hyperbaric medicine facilities knew the allocated treatment, but patients, clinicians, nurse practitioners, and other health-care professionals associated with patients' care were masked to treatment allocation. Primary endpoints were changes in the bowel component of the modified Inflammatory Bowel Disease Questionnaire (IBDQ) score and the IBDQ rectal bleeding score 12 months after start of treatment relative to baseline. The primary outcome was analysed in a modified intention-to-treat population, excluding patients who did not provide IBDQ scores within a predetermined time-frame. All patients have completed 12 months of follow-up and the final analysis is complete. The trial is registered with the ISRCTN registry, number ISRCTN86894066.

**Findings:**

Between Aug 14, 2009, and Oct 23, 2012, 84 participants were randomly assigned: 55 to hyperbaric oxygen and 29 to sham control. 75 (89%) participants received 40 pressure exposures, all participants returned the IBDQ at baseline, 75 (89%) participants returned the IBDQ at 2 weeks post-treatment, and 79 (94%) participants returned the IBDQ at 12 months post-start of treatment. Patients were excluded from analyses of co-primary endpoints if they had missing IBDQ scores for intestinal function or rectal bleeding at baseline or at 12 months. In an analysis of 46 participants in the active treatment group and 23 participants in the control group, we found no significant differences in the change of IBDQ bowel component score (median change from baseline to 12 months of 4 (IQR −3 to 11) in the treatment group *vs* 4 (−6 to 9) in the sham group; Mann-Whitney *U* score 0·67, p=0·50). In an analysis of 29 participants in the active treatment group and 11 participants in the sham group with rectal bleeding at baseline, we also found no significant differences in the change of IBDQ rectal bleeding score (median change from baseline to 12 months of 3 [1 to 3] in the treatment group *vs* 1 [1 to 2] in the sham group; *U* score 1·69, p=0·092). Common adverse events in both groups were eye refractive changes (three [11%] of 28 patients in the control group *vs* 16 [30%] of 53 patients in the treatment group), increased fatigue (three [11%] *vs* two [4%]), and ear pain (six [21%] *vs* 15 [28%]). Eight serious adverse events were reported in eight patients: two were reported in two patients in the control group (tonsillitis requiring surgery [grade 3]; recurrent cancer of the vulva [grade 4]) and six serious adverse events were reported in six patients in the treatment group (malignant spinal cord compression requiring surgery [grade 3]; malignant paraortic lymph node involvement requiring surgery [grade 3]; recurrence of vomiting and dehydration [grade 3]; diarrhoea and fever associated with *Campylobacter* infection [grade 3]; recurrence of abdominal pain, bloating, diarrhoea, and urinary tract infection [grade 3]; aneurysm [grade 4]), none of which were deemed treatment-related.

**Interpretation:**

We found no evidence that patients with radiation-induced chronic gastrointestinal symptoms, including those patients with rectal bleeding, benefit from hyperbaric oxygen therapy. These findings contrast with evidence used to justify current practices, and more level 1 evidence is urgently needed.

**Funding:**

Cancer Research UK and National Health Service (NHS) funding to the National Institute of Health Research Biomedical Research Centre at The Royal Marsden and the Institute of Cancer Research.

**Research in context**

**Evidence before this study**

Hyperbaric oxygen is widely used to treat chronic adverse effects of curative radiotherapy in long-term survivors of pelvic malignancy, especially those with rectal bleeding. We searched PubMed from Jan 1, 1970, to Dec 31, 2008, with the terms “clinical trials” AND “hyperbaric oxygen” AND “pelvic” OR “pelvis” OR “bowel” AND radiotherapy”. We identified 11 relevant publications, including reviews, relatively small case studies, and case reports. The results of a single randomised, sham-controlled trial from 2008 (HORTIS) reported significant clinical benefits for patients treated with hyperbaric oxygen 2 weeks post-treatment. A Cochrane intervention review from 2012 confirmed the retrospective nature of much research and detected no other level 1 evidence on which to base an assessment of this treatment modality for patients with chronic radiation-induced bowel dysfunction.

**Added value of this study**

The results of this double-blind, sham-controlled clinical trial fail to confirm earlier positive results of hyperbaric therapy for cancer survivors with chronic bowel dysfunction, including a subset of patients with rectal bleeding, after curative radiotherapy for pelvic malignancy, with a similarly sized minority of volunteers in each randomised group reporting some improvement in symptoms. This trial is only the second randomised study in this important patient population.

**Implications of all the available evidence**

The contribution of hyperbaric oxygen to the management of a growing population of long-term cancer survivors with severe restrictions on daily activities and impaired quality of life as a consequence of bowel injuries after curative radiotherapy for pelvic malignancy remains unclear and requires more evidence from well designed clinical trials.

## Introduction

More than 1 million patients worldwide are estimated to need curative radiotherapy for pelvic cancer annually, with up to a third of these patients subsequently developing chronic moderate or severe gastrointestinal symptoms.^1^ Hyperbaric oxygen has been used as a therapy for symptomatic patients for decades, yet the evidence to support the use of this therapy is based almost exclusively on non-randomised studies.^2^ The authors of a 2012 Cochrane intervention review^3^ identified a single well designed, controlled, randomised trial (HORTIS)^4^ that showed clinical benefit of hyperbaric oxygen therapy in patients with gastrointestinal symptoms after radiotherapy for cancers of the colon, endometrium, uterine corpus, uterine cervix, prostate, or rectum. We conducted a double-blind, randomised controlled trial (HOT2) to test long-term benefits of hyperbaric oxygen therapy in patients with chronic adverse effects of curative pelvic radiotherapy after failure of optimum medical therapy for symptoms of pelvic radiation disease.

## Methods

### Study design and participants

The HOT2 trial was a randomised, double-blind, sham-controlled phase 3 study involving ten UK hyperbaric medicine facilities registered with the British Hyperbaric Association (appendix p 2).

Eligible participants were men and women aged 18 years or older with at least grade 2 gastrointestinal symptoms in any category of the Late Effects Normal Tissue scoring system (LENT SOMA) for radiation injury or grade 1 gastrointestinal symptoms with intermittent symptoms attributed to radiotherapy for carcinoma of the rectum, prostate, testis, bladder, uterine cervix, uterine corpus, vagina, vulva, or ovary for at least 12 months before enrolment. Grade 2 symptoms defined by LENT SOMA are moderate, requiring only conservative treatment, whereas grade 3 symptoms are severe, having a substantial negative effect on daily activities, and necessitating more aggressive treatment.^5^ Participants were screened for eligibility if they presented with gastrointestinal symptoms such as onset or worsening of anal, rectal, atypical abdominal, or back pain; endoscopic evidence of anal, rectal, or sigmoid stricture; worsening of intestinal symptoms after months or years of stable symptoms; worsening of urinary symptoms; or new vaginal bleeding. Potentially eligible participants were assessed using a clinical algorithm^6^ to identify individuals with symptoms attributable to radiotherapy. Eligible patients could show no evidence of cancer recurrence, as assessed by magnetic resonance imaging of the pelvis, abdomen, and spine. Additional exclusion criteria included medical history of cancer recurrence, rectal surgery, previous hyperbaric oxygen therapy (except for treatment of decompression illness), exposure to bleomycin, claustrophobia, epilepsy, uncontrolled asthma, bullous lung disease, some types of ear surgery, and inability to equalise the middle ear. Individuals with a past history of prostate cancer had to have three serial measurements of serum prostate-specific antigen within the normal concentration range (less than 3 ng/mL for men aged 50–59 years, 4 ng/mL for men aged 60–69 years, 5 ng/mL for men 70 years or older).

Patients with symptoms attributed to radiotherapy entered a minimum 3-month period of optimum standard treatment, including antibiotic treatment for small bowel bacterial overgrowth, treatment of bile acid malabsorption,^7^ lifestyle advice, or several of these interventions, and were supervised by a gastroenterologist. Individuals were considered eligible for the study only if the 3-month period of optimal standard treatment was unsuccessful.

All patients provided written informed consent. We listed no criteria for removing a patient from the trial once written informed consent was gained. The study was approved by the MHRA (2008-002152-26) and the NRES Committee North East-York (08/H0903/40). The full case study report and trial protocol are available online.

### Randomisation and masking

Eligible participants were randomly assigned (2:1) to receive hyperbaric oxygen treatment or sham. Randomisation was arranged by a telephone call from the treating hyperbaric medicine facility to the Institute of Cancer Research Clinical Trials and Statistics Unit (ICR-CTSU). Randomisation was by computer-generated random permuted blocks (block size of nine and 12), and participants were stratified by centre. Computer-generated lists were used to allocate patients within a block. To deliver the correct treatment, only engineers and technicians operating the hyperbaric chamber were informed of the allocated treatment by the trials office, and care was taken to ensure that patients, clinicians, nurse practitioners, and other health-care professionals associated with patients' care remained masked to treatment allocation. The most important precaution was to disallow any non-trial patient sharing the chamber with a trial patient.

### Procedures

Participants attended the participating hyperbaric oxygen medicine facility most convenient for them, where they were assessed for suitability for hyperbaric oxygen therapy. Patients in the hyperbaric oxygen therapy group received 40 pressure exposures at 2·4 atmospheres of absolute pressure (ATA; 243 kPa) breathing 100% oxygen for 90 min (including 5-min air breaks at 30-min intervals), whereas patients in the control group received 40 pressure exposures at 1·3 ATA (131 kPa) breathing 21% oxygen (ie, air) for 90 min with two simulated 5-min air breaks. We aimed to deliver the pressure exposures once a day for 5 days per week for 8 weeks for a total of 40 pressure exposures. Additional treatments were delivered beyond the 8-week timeframe if any scheduled sessions were missed. Dose reductions were not permitted.

Participants were asked to complete the modified Inflammatory Bowel Disease Questionnaire (IBDQ)^8^ and the European Organisation for Research and Treatment of Cancer (EORTC) C30 core quality of life questionnaire (QLQ-C30) and CR38 colorectal module (QLQ-CR38)^9^, ^10^ at baseline, 2 weeks after end of treatment, and again at 3 months, 6 months, 9 months, and 12 months after start of treatment. At each timepoint, patients were asked to base their responses on symptoms experienced within the previous 2 weeks. The IBDQ bowel function component (panel) was adopted on the basis of previous application for the characterisation of chronic gastrointestinal morbidity after pelvic radiotherapy in a comparable population of former patients.^8^, ^12^ Late radiation-induced adverse effects were clinically assessed within 2 weeks of treatment completion and again at 12 months after start of treatment and were based on the LENT SOMA intestinal and rectal scales of radiation injury (version 2) and 11 questions selected from the Common Terminology Criteria for Adverse Events (CTCAE) gastrointestinal scale (version 4), which were considered most relevant to the study population.^5^, ^13^ Telephone interviews were substituted for the minority of patients unable to attend appointments at the Royal Marsden as per protocol.

### Outcomes

The two primary clinical endpoints of the study were the change in gastrointestinal symptoms score using the IBDQ and the change in rectal bleeding score (Question 22) in the IBDQ between baseline and 12 months (panel). Secondary clinical endpoints were adverse effects (bowel dysfunction) assessed according to LENT SOMA scales of radiation injury, clinical assessments of gastrointestinal symptoms according to the 11 questions selected from the CTCAE gastrointestinal scale (version 4), and patient self-assessments of quality of life recorded by the EORTC QLQ-C30 core questionnaire and QLQ-CR38 colorectal module between baseline and 12 months.

### Statistical analysis

The sample size was calculated on the basis of the bowel component of the modified IBDQ primary endpoint. On the basis of results from a previous study,^14^ we considered a reduction in IBDQ bowel component score of 7 (SD 10) from baseline to 12 months to be clinically relevant. To detect this minimum change at a two-sided significance level of 5% and an estimated power of 80%, we planned to enrol 75 evaluable patients. During the recruitment phase of the trial (February, 2012), the independent data monitoring committee agreed that the significance level of 5% could be split to allow additional analyses in patients reporting rectal bleeding in the IBDQ at baseline. We estimated that 75 evaluable patients would allow us to detect a difference in IBDQ bowel symptom score of 7·5 with 80% power at a two-sided significance level of 3%. On the basis of the assumption that 30 of 75 patients would report grade 2–4 rectal bleeding at baseline on the LENT SOMA Management Scale, corresponding to a score of 1–5 on the IBDQ rectal bleeding scale, this subgroup would allow us to detect a difference of 70% of patients showing any improvement in rectal bleeding (10% in the control group, 80% in the hyperbaric oxygen therapy group) with 80% power at a two-sided significance level of 2%.

Analysis of primary endpoints was by modified intention-to-treat, which included analysis of data from forms returned by patients within timeframes agreed to by the independent data monitoring committee. All patients who received any treatment (hyperbaric oxygen therapy or sham) were included in the safety population. Forms were processed as follow-up assessments according to the period that had elapsed between start of treatment and time of completion (table 1). IBDQ questions are scored from 1 to 7 with a low score indicating poorer function or worse symptoms. The bowel component is made up of ten questions, and we used all ten items in the bowel component of the modified IBDQ to analyse overall bowel function and analysed rectal bleeding using the single rectal bleeding question in the modified IBDQ. The difference in change from baseline to 12 months between the two study groups was analysed using the Mann-Whitney *U* test due to the non-normality of the data. We planned sensitivity analyses of the primary endpoints and the LENT SOMA secondary endpoint in the population of patients who were registered into the study and returned IBDQ forms, irrespective of timelines (intention-to-treat), and in the per-protocol population, which included all patients registered into the study who received at least 32 pressure exposures within a 10-week period. These sensitivity analyses excluded individuals who received less than three treatments per week for at least 2 weeks or who missed five consecutive treatments.

For the comparison of change in LENT SOMA scores from baseline to 12 months for rectum and intestine (secondary endpoints; table 2) between the active treatment and control groups, we scored individual symptoms within each of three LENT SOMA descriptors (subjective, objective, management) using a four-point scale (with high scores denoting worse symptoms) and summed these scores to develop overall subjective, objective, and management scores for each anatomical site (rectum and intestine). We did no formal statistical analyses of other secondary endpoints (CTCAE scales, EORTC QLQ-C30, and QLQ-CR38^10^), although the descriptive results were used to strengthen interpretation of changes in the primary endpoints. In an exploratory analysis we tested for a difference in the proportion of patients reporting an improvement in rectal bleeding at 12 months between the two study groups using all available questionnaires (ie, the CTCAE rectal bleeding questions, rectal LENT SOMA objective and management scores, intestinal LENT SOMA management score, and EORTC QLQ-CR38 question 59 “Have you had blood with your stools?”). Patients reporting no rectal bleeding at baseline on an individual scale were excluded from the analysis of that scale. We did two exploratory subgroup analyses of the primary endpoints; one analysis considered the group of patients who received radiotherapy 1–5 years before randomisation, and the other considered the group of patients whose trial treatment was delivered by hood (or monochamber).

We used Stata version 13 for all statistical analyses. The trial is registered with the ISRCTN registry, number ISRCTN86894066.

### Role of funding source

The funder had no role in study design, data collection, data analysis, data interpretation, or writing of the report. The corresponding author had full access to all the data and had final responsibility for the decision to submit for publication.

## Results

Between Aug 14, 2009, and Oct 23, 2012, 241 patients were given a rigorous initial assessment followed by a 3-month period of optimised medication. 84 participants were considered eligible for trial entry and were randomly assigned to treatment with hyperbaric oxygen (active treatment group; n=55) or with sham control (control group; n=29; figure). The trial ended when all patients had been followed up for 12 months from start of treatment; the final data were collected on Oct 28, 2013. Median follow-up was 13·2 months (IQR 12·4–14·2). Baseline characteristics of the study population are summarised in table 3. There was a small imbalance in the proportion of patients reporting a medical history of rectal bleeding at trial entry, but this was not reflected in the baseline IBDQ or LENT SOMA scales (online case study report). Two-thirds of participants had faecal frequency, incontinence, or both, symptoms that suggest injury to the colon as well as rectum, and a similar proportion reported rectal bleeding. 75 (89%) participants received all 40 planned pressure exposures, and nine (11%) patients received 38 exposures or less (one patient received 38 exposures, one patient received 31 exposures, one patient received 18 exposures, one patient received 11 exposures, one patient received four exposures, one patient received two exposures, and three patients received no exposures). Table 4 details the number of IBDQ and LENT SOMA assessment forms returned within prespecified timeframes.

We found no significant differences in the improvement of overall bowel function (Mann-Whitney *U* score 0·67; p=0·50) or rectal bleeding (*U* score 1·69; p=0·092) after 12 months between randomised groups (table 5). Of the patients in the modified intention-to-treat population who reported slight increase in frequency or worse rectal bleeding on IBDQ at baseline, ten (67%) of 15 patients in the control group and 26 (74%) of 35 patients in the treatment group reported an improvement of at least 1 point in the IBDQ rectal bleeding score at 12 months (absolute difference 7·6% [95% CI −20·3 to 35·5]; p=0·58; appendix p 2). Analysis of the IBDQ baseline data did not show imbalances in the pattern or severity of symptoms between treatment groups (figure and appendix p 1).

Sensitivity analyses of both primary endpoints, including all data returned for the 12-month timepoint irrespective of time of return, showed that the difference in change from baseline to 12 months between the two study groups was consistent with the modified intention-to-treat analysis (*U* score 0·71 [p=0·48] for overall bowel function; *U* score 2·06 [p=0·040] for rectal bleeding). Per-protocol analyses of the primary endpoints were also consistent with the modified intention-to-treat analysis (*U* score 0·94 [p=0·35] for overall bowel function; *U* score 1·44 [p=0·15] for rectal bleeding; appendix p 3).

Both treatment groups had a non-significant decrease in subjective LENT SOMA scores for rectum and intestine indicative of an improvement in symptoms (table 6). Sensitivity analyses including all data irrespective of specified timelines gave similar results (*U* score 1·62 [p=0·11] for rectal LENT SOMA scores; *U* score −1·41 [p=0·16] for intestinal LENT SOMA scores). Planned descriptive analysis of changes in CTCAE grades at baseline, 2 weeks post-treatment, and at 12 months also did not show differences between the treatment groups (appendix p 4). In view of these negative results, we did not report the planned descriptive analyses of the EORTC QLQ-C30 and QLQ-CR38 since they could not affect the interpretation or conclusions of the trial.

Exploratory analysis comparing patient-reported rectal bleeding obtained from the IBDQ questionnaire with scores from other scales including the CTCAE rectal bleeding, rectal LENT SOMA objective and management, intestinal LENT SOMA management, and EORTC QLQ-CR38 questionnaires were in line with those obtained using IBDQ with the exception of the rectal LENT SOMA management score (appendix p 2). Five (100%) of five patients in the control group reported an improvement in rectal bleeding LENT SOMA Management score compared with four (31%) of 13 patients in the hyperbaric oxygen therapy group. Exploratory subgroup analyses of patients who completed radiotherapy 1–5 years before entering the study did not show any difference in IBDQ scores between the two groups (*U* score 0·59 [p=0·56] for overall bowel function; *U* score 1·57 [p=0·12] for rectal bleeding). Exploratory subgroup analysis in patients receiving treatment using a hood or monochamber showed no difference in overall bowel function but did suggest a difference in rectal bleeding (*U* score −0·31 [p=0·76] for overall bowel function; *U* score 2·9 [p=0·004] for rectal bleeding).

We analysed toxic effects in the safety population, which included the 81 patients who received at least one treatment (53 in the hyperbaric oxygen therapy group and 28 in the sham control group). Treatment-emergent toxic effects were reported for 41 (51%) of 81 patients receiving at least one treatment in either treatment group. The most commonly reported adverse events were eye refractive change, including myopia (three [11%] of 28 patients in the control group *vs* 16 [30%] of 53 patients in the treatment group), increased fatigue or tiredness (three [11%] *vs* two [4%]), and ear pain or barotrauma (six [21%] *vs* 15 [28%]). Eight serious adverse events were reported in eight patients: two were reported in two patients in the control group (tonsillitis requiring surgery [grade 3]; recurrent cancer of the vulva [grade 4]) and six serious adverse events were reported in six patients in the treatment group (malignant spinal cord compression requiring surgery [grade3]; malignant paraortic lymph node involvement requiring surgery [grade 3]; recurrence of vomiting and dehydration [grade 3]; diarrhoea and fever associated with *Campylobacter* infection [grade 3]; recurrence of abdominal pain, bloating, diarrhoea, and urinary tract infection [grade 3]; aneurysm [grade 4]). No reported adverse event was considered related to treatment. One patient had an improvement in eyesight during treatment. Only two of the patients who stopped treatment early did so for reasons related to treatment (anxiety). No treatment-related deaths were noted.

## Discussion

Despite some clinical evidence and plausible pathophysiological mechanisms justifying an expectation of therapeutic effect of hyperbaric oxygen therapy, the HOT2 trial results detected no clinically relevant benefit of hyperbaric oxygen therapy in individuals with a wide range of chronic gastrointestinal dysfunction, including rectal bleeding, after curative radiotherapy for pelvic malignancy. The modified IBDQ was adopted to assess the primary outcome in HOT2, given its successful application in characterising patients with gastrointestinal dysfunction after pelvic radiotherapy.^6^, ^8^, ^11^, ^15^, ^16^, ^17^ None of the exploratory analyses using other instruments to measure rectal bleeding, including LENT SOMA, CTCAE, and EORTC, suggested any clinical benefit of hyperbaric oxygen.

Pelvic radiation syndrome describes a range of physiological disorders that often take a remittent course and are best characterised by investigation according to structured algorithms before treatment.^6^ The symptoms include pain, bloating, flatulence, diarrhoea, urgency, faecal incontinence, and rectal bleeding. Histologically, progressive obliterative endarteritis is a classic feature and ischaemic atrophy is an important element of the pathophysiology, but direct radiation effects on other tissue elements, including epithelia, also contribute to symptoms.^18^ The tissues rendered ischaemic by vascular atrophy do not share the steep oxygen gradients that stimulate angiogenesis in acute surgical wounds unless these gradients are artificially introduced.^19^ In studies of animal and human skin,^20^, ^21^ hyperbaric oxygen therapy has been shown to restore virtually normal small vessel density and transcutaneous oxygen tension after high-dose radiotherapy, an effect that peaked after 20–30 treatments in human beings. The proposed therapeutic mechanisms include marrow stem-cell mobilisation and consequent vasculogenesis, although our results do not suggest that these processes, if activated by hyperbaric oxygen, were of therapeutic value.^22^

Our trial results are inconsistent with a long history of striking anecdotes and reviews of non-randomised studies.^2^, ^23^, ^24^, ^25^ A Cochrane intervention review^3^ identified two randomised trials testing hyperbaric oxygen in patients with chronic gastrointestinal symptoms after pelvic radiotherapy, but the analysis was restricted to the HORTIS trial^4^ because of a high risk of bias identified in the other study. The HORTIS trial randomly assigned 150 patients from Mexico, Turkey, South Africa, and Australia with a 3-month or longer medical history of radiation proctitis to breathe air at 1·1 ATA (sham group) or 100% oxygen at 2·0 ATA (active treatment group) for 90 min for 30 sessions within 6–8 weeks, with an additional ten sessions depending on individual responses. Improvements in the LENT SOMA score (primary endpoint) were found in 120 evaluable patients with radiation proctitis; patients in the active treatment group recording significantly lower average scores than patients in the sham group (p=0·015), with an estimated difference of 1·93 points (95% CI 0·38–3·48). The HORTIS investigators also reported a significant benefit of hyperbaric oxygen in patients with bowel bother, a group of symptoms that include faecal incontinence, faecal urgency, and pain. The authors of the Cochrane review interpreted these results as non-significant and sensitive to randomised patients excluded from primary analysis but concluded that HORTIS supported the continued use of hyperbaric oxygen for patients with radiation proctitis.

It is unclear why the results of HOT2 fail to reproduce the HORTIS^4^ findings. Although a single-centre study in terms of patient referral and selection, HOT2 trial participants were treated at one of ten UK-registered hyperbaric facilities. Patient selection was unusually rigorous, including assessment by a gastroenterologist specialised in radiation enteropathy and a 3-month run-in period of optimised oral drug treatment to ensure that eligible patients had radiation-induced symptoms that could not be controlled by standard measures. The trial population is considered representative of patients with radiation enteropathy in terms of their symptoms, although patients with severe faecal incontinence or transfusion-dependent rectal bleeding are likely to be under-represented, the former being too restricted to leave their homes and the latter considered too seriously at risk to be considered by their primary physicians for entry into a trial with a sham treatment option. We assessed 20 characteristics relating to bowel dysfunction at baseline, and despite small imbalances in the proportion of patients with a medical history of rectal bleeding (23 [79%] of 29 patients in the control group *vs* 34 [62%] of 55 patients in the treatment group), this imbalance did not apply to baseline IBDQ rectal bleeding scores analysed as the primary endpoint. In other respects, symptom duration in HOT2, at a median 3·7 years (IQR 2·4–6·8) post-radiotherapy, is consistent with that of the HORTIS population, and HOT2 patient characteristics are well balanced between randomised groups.

In a literature review^26^ of ten retrospective studies published between 1960 and 2004 reporting favourable results of hyperbaric oxygen therapy for patients with radiation proctitis, patients had an average of 24 treatments each. The randomised, sham-controlled HORTIS trial^4^ reported beneficial clinical effects of hyperbaric oxygen after 30–40 treatments. Hence, the 40 treatments used in HOT2 can be considered an appropriate test of hyperbaric oxygen. Compliance with treatment in HOT2 was reasonably high, with 75 (88%) of 84 patients eligible for inclusion in the intention-to-treat population, as required by the analysis plan. Post-hoc analyses in the per-protocol population failed to detect any treatment effect. Pre-trial investigations designed to exclude patients with residual malignant disease ensured that only three patients developed cancer recurrence while participating in the study.

Other relevant points of difference between the HORTIS^4^ and HOT2 trials include the immediate post-treatment timepoint for the primary analysis in HORTIS, compared with the primary analysis at 12 months post-treatment in HOT2. Exploratory analyses of the 2-week post-treatment effects in HOT2 showed no difference between randomised groups for any of the primary or secondary endpoints. This included change in total LENT SOMA score, which was the primary endpoint analysed by the HORTIS investigators. Unlike HOT2, in which we analysed the primary endpoint in a modified intention-to-treat population, the primary analysis in HORTIS excluded 30 of 150 randomised patients who did not complete the treatment protocol (plus one patient lost to follow-up), although unplanned analyses of the outcomes of clinical assessments by intention-to-treat were also consistent with a beneficial effect of hyperbaric oxygen in HORTIS. In other exploratory analyses of HOT2 endpoints (including subgroup analysis of patients completing radiotherapy 1–5 years before randomisation and subgroup analysis of patients receiving treatment by hood or hyperbaric chamber rather than mask), we could not identify variables that might explain differences in reported outcomes between these two trials. Randomisation appears to have resulted in a reasonably even distribution of patient characteristics between the treatment and control groups in our study, to the extent that the total effect of these covariates would not be expected to mask any effect of hyperbaric oxygen. The very small number of patients with transfusion-dependent rectal bleeding in this study prevents us from commenting on the use of hyperbaric oxygen therapy for patients referred for this potentially life-threatening complication.

Our trial was designed to have a power of 80%; with 69 evaluable patients, we had a power of about 75% to detect a difference of the magnitude expected in the first of the primary endpoints. We did not detect a clinically or statistically significant clinical benefit of hyperbaric oxygen therapy for patients with chronic gastrointestinal dysfunction, including rectal bleeding, after pelvic radiotherapy. The findings contrast with previous reports, highlighting an urgent need for more level 1 evidence to determine with confidence whether hyperbaric oxygen therapy can be recommended as a standard of care for this group of patients.

## Figures and Tables

**Figure:**
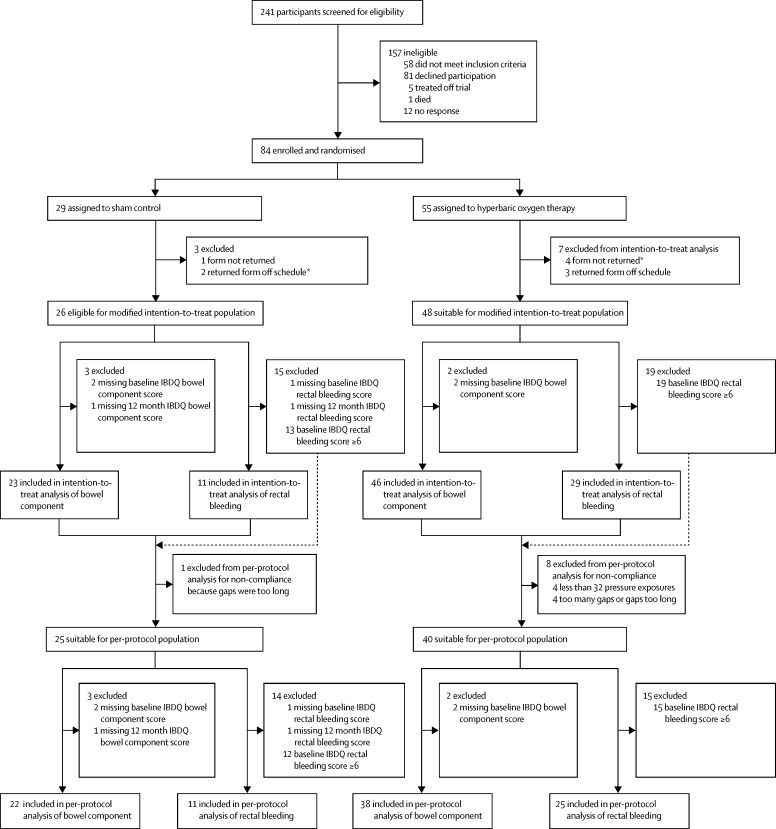
Trial profile IBDQ=modified Inflammatory Bowel Disease Questionnaire. *Includes one patient in the control group and two patients in the hyperbaric oxygen therapy group who received no treatment.

**Table 1:** Classification of patient case report forms according to time of return after start of treatment

	**Form to be processed as**
Completed after 2-week assessment and less than 4·5 months after start of treatment	3-month assessment
Completed 4·5–7·5 months after start of treatment	6-month assessment
Completed 7·5–10·5 months after start of treatment	9-month assessment
Completed 10·5–14 months after start of treatment	12-month assessment
Completed more than 14 months after start of treatment	Exclude forms

**Table 2:** Subjective parameter score of the 4-point rectal and intestinal LENT SOMA scoring scales^5^

	**Score of 1**	**Score of 2**	**Score of 3**	**Score of 4**
Rectal*				
Stool frequency	2–4 per day	5–8 per day	>8 per day	Uncontrolled diarrhoea
Sphincter control	Occasional	Intermittent	Persistent	Refractory
Pain	Occasional and minimal	Intermittent and tolerable	Persistent and intense	Refractory and excruciating
Tenesmus	Occasional urgency	Intermittent urgency	Persistent urgency	Refractory
Mucosal loss	Occasional	Intermittent	Persistent	Refractory
**Intestinal**†				
Stool frequency	2–4 per day	5–8 per day	>8 per day	Refractory diarrhoea
Stool consistency	Bulky	Loose	Mucous, dark, watery	··
Pain	Occasional and minimal	Intermittent and tolerable	Persistent and intense	Refractory/rebound
Constipation	3–4 per week	Only twice per week	Only once per week	No stool in 10 days

LENT SOMA=Late Effects in Normal Tissues Subjective, Objective, Management and Analytic scales.

* The possible range of summed results for the five questions is 0–20, where 0 indicates that no symptoms are present and 20 represents the worst possible symptomatology.

† The possible range of summed results for the four questions is 0–15, where 0 indicates that no symptoms are present and 15 represents the worst possible symptomatology (there is no grade 4 stool consistency).

**Table 3:** Patient characteristics at pretrial eligibility assessments

	**Sham control (n=29)**	**Hyperbaric oxygen (n=55)**
**Age**		
Mean	62·0 (11)	62·3 (11)
Median	63·7 (53·6–69·9)	63·7 (53·9–71·2)
Range	37·3–79·3	34·5–80·9
**Sex**		
Male	14 (48%)	23 (42%)
Female	15 (52%)	32 (58%)
**Origin of cancer**		
Prostate	12 (41%)	21 (38%)
Anus	4 (14%)	4 (7%)
Vagina	3 (10%)	1 (2%)
Cervix	5 (17%)	17 (31%)
Uterus	3 (10%)	8 (15%)
Other*	2 (7%)	4 (7%)
**Medical history**		
Back pain	3 (10%)	7 (13%)
Bloating	18 (62%)	30 (55%)
Constipation	5 (17%)	5 (9%)
Cramps or abdominal pain	14 (48%)	38 (69%)
Diarrhoea	14 (48%)	30 (55%)
Faecal incontinence	19 (66%)	35 (64%)
Frequency	18 (62%)	38 (69%)
Mucus discharge	10 (34%)	21 (38%)
Nausea	4 (14%)	13 (24%)
Other	6 (21%)	8 (15%)
Rectal bleeding	23 (79%)	34 (62%)
Rectal or perineal pain	8 (28%)	10 (18%)
Steatorrhoea	1 (3%)	10 (18%)
Subacute obstructive symptoms	3 (10%)	14 (25%)
Tenesmus	18 (62%)	35 (64%)
Unable to differentiate need to defecate or pass urine	1 (3%)	2 (4%)
Unable to differentiate solid or liquid stool	6 (21%)	11 (20%)
Urgency	20 (69%)	48 (87%)
Weight loss	2 (7%)	10 (18%)
Wind	17 (59%)	39 (71%)
**Time since pelvic radiotherapy (years)**		
Median	3·9 (2·5–5·7)	3·5 (2·3–9·7)
Range	1·5–21·2	1·2–34·0

Data are n (%) or median (IQR).

* Others were anal canal (n=1) and vulva (n=1) in the control group and retroperitoneum (n=1), pelvis (n=1), rectum (n=1), and bladder (n=1) in the hyperbaric oxygen therapy group.

**Table 4:** Overall returns of IBDQ and LENT SOMA assessment forms and those returned within prespecified timeframes

	**Baseline**	**2 weeks**	**3 months**	**6 months**	**9 months**	**12 months**
IBDQ forms returned	84	75	79	78	78	79
IBDQ forms returned*	84	75	68	76	74	74
Rectal LENT SOMA	84	78	··	··	··	79
Rectal LENT SOMA*	84	78	··	··	··	72
Intestinal LENT SOMA	84	78	··	··	··	79
Intestinal LENT SOMA*	84	78	··	··	··	72
CTCAE	83	78	··	··	··	79
CTCAE*	83	78	··	··	··	72
QLQ-C30	84	··	77	78	78	79
QLQ-C30*	84	··	65	76	75	74
QLQ-CR38	84	··	77	78	78	79
QLQ-CR38*	84	··	65	76	74	74

IBDQ=Inflammatory Bowel Disease Questionnaire. LENT SOMA=Late Effects in Normal Tissues Subjective, Objective, Management and Analytic scales. CTCAE=Common Terminology Criteria for Adverse Events.

* Includes only forms returned within the prespecified permissible timeframes detailed in table 1.

**Table 5:** Median changes in the IBDQ bowel function component and IBDQ rectal bleeding scores from baseline to 12 months in patients assessed within 10–14 months

	**Median score at baseline (IQR)**		**Median score at 12 months (IQR)**		**Median change from baseline to 12 months (IQR)**		**Mann-Whitney test***U***score**	**p value**
	Sham	Hyperbaric oxygen	Sham	Hyperbaric oxygen	Sham	Hyperbaric oxygen		
Bowel function*	51 (44 to 59)	48 (42 to 52)	53 (40 to 59)	51 (36 to 62)	4 (−6 to 9)	3·5 (−3 to 11)	0·67	0·50
Rectal bleeding†	3 (2 to 4)	3 (2 to 4)	4 (2 to 6)	6 (3 to 7)	1 (1 to 2)	3 (1 to 3)	1·69	0·092

Positive changes indicate higher median Inflammatory Bowel Disease Questionnaire (IBDQ) scores, which signify improvement in symptoms.

* Analysis included 23 patients in the sham control group and 46 patients in the hyperbaric oxygen treatment group.

† Analysis included 11 patients in the sham control group and 29 patients in the hyperbaric oxygen treatment group. 40 (47%) of 84 patients scored grade 1–5 (clinically significant) rectal bleeding on the IBDQ bowel function component, compared with 57 (68%) patients reporting any rectal bleeding in their medical history (panel). 46 (55%) patients had grade 1–3 rectal bleeding at baseline according to Common Terminology Criteria for Adverse Events scoring system, and 47 (59%) patients had grade 1–3 rectal bleeding in response to EORTC QLQ-CR38 Question 59.

**Table 6:** Median changes in LENT SOMA aggregate parameter scores for rectum and intestine from baseline to 12 months in patients assessed within 10–14 months

	**Median score at baseline (IQR)**		**Median score at 12 months (IQR)**		**Median change from baseline to 12 months (IQR)**		**Mann-Whitney test***U***score**	**p value**
	Sham (n=26)	Hyperbaric oxygen (n=46)	Sham (n=26)	Hyperbaric oxygen (n=46)	Sham (n=26)	Hyperbaric oxygen (n=46)		
Rectum	6 (5 to 8)	6 (4 to 8)	4·5 (2 to 8)	5 (3 to 8)	−1·5 (−4 to 0)	−1 (−2 to 1)	1·56	0·12
Intestine	2·5 (1 to 4)	4 (2 to 5)	1 (1 to 4)	2·5 (1 to 4)	0 (−1 to 1)	0 (−2 to 0)	−1·30	0·20

High scores indicate worse symptoms; a negative change indicates a lower score at 12 months, signifying improvement in function. LENT SOMA=Late Effects in Normal Tissues Subjective, Objective, Management and Analytic scale. 46 (55%) patients had grade 1–4 rectal bleeding at baseline as assessed by the LENT SOMA scale.

## Panel Bowel function component of the modified Inflammatory Bowel Disease Questionnaire (IBDQ)11

Question 1: Have you had your bowel open?

Question 5: Have you had loose bowel movements?

Question 9: Have you been troubled by pain in your bottom?

Question 13: Have you had cramp in tummy or bottom?

Question 17: Have you passed a large amount of gas?

Question 20: Have you been troubled by bloating?

Question 22: Have you had a problem with bleeding from your bottom?

Question 24: Have you felt like you need to have your bowel open but nothing happens?

Question 26: Have you been troubled by accidental soiling?

Question 29: Have you felt disgusted about your bowel problems?

Each question is linked to the following response options on a 7-point graded scale: 1=more than ever before; 2=extremely frequently; 3=very frequently; 4=moderate increase in frequency; 5=some increase in frequency; 6=slight increase in frequency; 7=normal/not at all. The possible range of summed results for the 10 questions is 10–70, where 10 represents the most severe, and 70 the least severe, levels of effect, this metric represents a co-primary endpoint. Question 22, analysed separately, is the second co-primary endpoint.
