# Supplementary material for: Hyperbaric oxygen for patients with chronic bowel dysfunction after pelvic radiotherapy (HOT2): a randomised, double-blind, sham-controlled phase 3 trial
Source: Lancet Oncol. 2016 Feb;17(2):224–33. doi: 10.1016/S1470-2045(15)00461-1 (PMC4737893; doi:10.1016/S1470-2045(15)00461-1)

THE LANCET Oncology

Supplementary appendix

This appendix formed part of the original submission and has been peer reviewed. We post it as supplied by the authors.

Supplement to: Glover M, Smerdon GR, Andreyev HJ, et al. Hyperbaric oxygen for patients with chronic bowel dysfunction after pelvic radiotherapy (HOT2): a randomised, double-blind, sham-controlled phase 3 trial. *Lancet Oncol* 2015; published online Dec 15. http://dx.doi.org/10.1016/S1470-2045(15)00461-1.

Supplementary Tables and Figures to be added to the internet

Table S1: IBDQ raw scores at baseline and 12 months

		Control		HBO		Total	
	**IBDQ rectal bleeding score**	**N**	**%**	**N**	**%**	**N**	**%**
**Baseline**	More than ever before	3	10.70%	8	14.50%	11	13.30%
	Extremely frequently	3	10.70%	6	10.90%	9	10.80%
	Very frequently	4	14.30%	8	14.50%	12	14.50%
	Moderate increase in frequency	3	10.70%	6	10.90%	9	10.80%
	Some increase in frequency	1	3.60%	5	9.10%	6	7.20%
	Slight increase in frequency	5	17.90%	7	12.70%	12	14.50%
	Not at all / normal	9	32.10%	15	27.30%	24	28.90%
	Total	28	100.00%	55	100.00%	83	100.00%
**12 months**	More than ever before	1	4.00%	4	8.30%	5	6.80%
	Extremely frequently	2	8.00%	1	2.10%	3	4.10%
	Very frequently	4	16.00%	3	6.30%	7	9.60%
	Moderate increase in frequency	3	12.00%	2	4.20%	5	6.80%
	Some increase in frequency	2	8.00%	4	8.30%	6	8.20%
	Slight increase in frequency	4	16.00%	12	25.00%	16	21.90%
	Not at all / normal	9	36.00%	22	45.80%	31	42.50%
	Total	25	100.00%	48	100.00%	73	100.00%

N.B. table includes all patients in ITT population

Table S2: Exploratory endpoints of rectal bleeding. 2 sample tests of proportion of patients with improvement in symptoms between baseline and 12 months

	Control<br>n (%)	HBO<br>n (%)	Absolute difference<br>% (95% CI)	P-value
IBDQ rectal bleeding question	10/15 (66.7%)	26/35 (74.3%)	7.6% (-20.3%, 35.5%)	0.58
Rectal LENT SOMA (objective)	12/16 (75.0%)	14/23 (60.9%)	-14.1% (-43.3%, 15.0%)	0.36
Rectal LENT SOMA (management)	5/5 (100.0%)	4/13 (30.8%)	-69.2% (-94.3%, -44.1%)	0.0085
CTCAE rectal bleeding	12/17 (70.6%)	13/22 (59.1%)	-11.5% (-41.4%, 18.4%)	0.46
EORTC QLQ-CR38 question 59	8/18 (44.4%)	17/29 (58.6%)	14.2% (-14.9%, 43.3%)	0.34

N.B. includes all patients included in the ITT population for whom rectal bleeding was present at baseline (according to each of the scales separately)

Table S3: Recruitment by centre

Hyperbaric treatment centre	n
London Diving Chamber, Hospital of St John and St Elizabeth, London	**24**
Hyperbaric Medicine Unit, St Richard's Hospital, Chichester	**15**
Whipps Cross University Hospital, Leytonstone, London	**11**
South Wales Hyperbaric Medical Centre (DDRC), Cardiff	**7**
North of England Medical and Hyperbaric Services, Spire Hull & East Riding Hospital, Kingston-upon-Hull	**6**
DDRC Healthcare, Plymouth	**6**
The Diver Clinic, Poole	**5**
Midlands Diving Chamber, Hospital of St Cross, Rugby	**4**
East of England Hyperbaric Unit, Lowestoft Road, Great Yarmouth	**3**
North West Emergency Recompression Unit, Murrayfield Hospital, Wirral	**3**
Total	**84**

Table S4: Per protocol analysis of the primary endpoint

Primary endpoint 1: Bowel function component of the modified IBDQ					
	Median (IQR) score at baseline	Median (IQR) score at 12 months	Median (IQR) change from baseline to 12m	Mann-Whitney test U Score	P-value
Control (n=22)	51 (44, 59)	51.5 (40, 59)	3.5 (-6, 9)	0.94	0.35
HBO (n=38)	48 (42, 51)	52 (36, 62)	4 (-2, 11)		
**Primary endpoint 2: rectal bleeding score from the modified IBDQ**					
	Median (IQR) score at baseline	Median (IQR) score at 12 months	Median change (from baseline to 12m)	Mann-Whitney test U Score	P-value
Control (n=11)	3 (2, 4)	4 (2, 6)	1 (1, 2)	1.44	0.15
HBO (n=25)	3 (2, 4)	6 (3, 7)	3 (0, 3)		

Table S5: Exploratory analysis of LENT SOMA scales

Change in rectal LENT SOMA total score from baseline to 2 weeks					
	Median (IQR) score at baseline	Median (IQR) score at 2 weeks	Median (IQR) change from baseline to 2 weeks	Mann-Whitney test U Score	P-value
Control (n=28)	**9.5 (8, 14.5)**	**8.5 (5.5, 14.0)**	**-2 (-3, 0)**	**0.35**	**0.72**
HBO (n=50)	**10 (7, 13)**	**8 (4, 12)**	**-2 (-3, 1)**		

Table S6: Exploratory analysis of primary endpoint according to treatment with hood, monochamber or mask.

Bowel function in patients treated with a hood or monochamber					
	Median (IQR) score at baseline	Median (IQR) score at 12 months	Median (IQR) change from baseline to 12m	Mann-Whitney test U Score	P-value
Control (n=14)	53 (43, 56)	54.5 (33, 59)	4 (1, 9)	-0.31	0.76
HBO (n=29)	48 (44, 54)	55 (36, 63)	2 (-7, 11)		
**Bowel function in patients treated with a mask**					
	Median (IQR) score at baseline	Median (IQR) score at 12 months	Median change (from baseline to 12m)	Mann-Whitney test U Score	P-value
Control (n=9)	51 (47, 59)	50 (47, 55)	1 (-8, 4)	1.59	0.11
HBO (n=17)	46 (35, 51)	49 (46, 55)	8 (1, 13)		

Figure S1: Maximum CTCAE toxicities at each timepoint

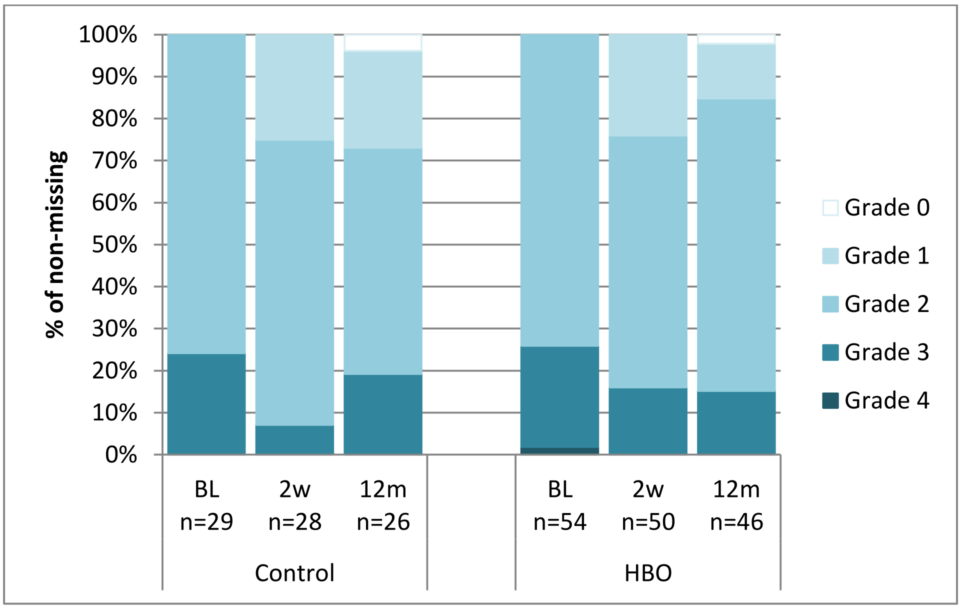

Figure S2: CTCAE rectal bleeding grade at each timepoint

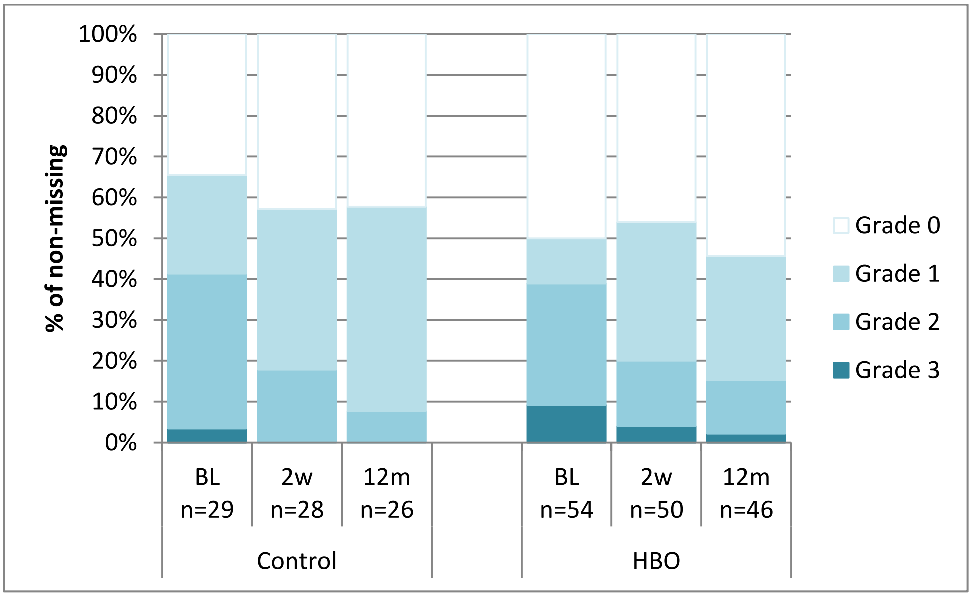

Supplement: Supplementary appendix [file mmc1.pdf]
